# A pan-cancer analysis of the oncogenic role of leucine zipper protein 2 in human cancer

**DOI:** 10.1186/s40164-022-00313-x

**Published:** 2022-09-15

**Authors:** Dechao Feng, Xu Shi, Weizhen Zhu, Facai Zhang, Dengxiong Li, Ping Han, Qiang Wei, Lu Yang

**Affiliations:** Department of Urology, Institute of Urology, West China Hospital, Sichuan University, Guoxue Xiang #37, Chengdu, 610041 Sichuan People’s Republic of China

## Abstract

In this study, we aimed to perform a pan-cancer analysis of leucine zipper protein 2 (LUZP2). A standardized TCGA pan-cancer dataset was downloaded. Differential expression, clinical prognosis, genetic mutations, immune infiltration, epigenetic modifications, tumor stemness and heterogeneity were analyzed. We conducted all analyses through software R 3.6.3 and its suitable packages. Compared to normal samples, we observed that the LUZP2 mRNA expression was significantly upregulated in LGG, PRAD, LUSC and downregulated in KIRC and other eleven cancer species patients. In terms of overall survival, low-expression of LUZP2 was significantly associated with poor prognosis in lower grade glioma (LGG), lung squamous cell carcinoma (LUSC), kidney renal clear cell carcinoma (KIRC) and prostate adenocarcinoma (PRAD). For progression-free survival, we observed that downregulation of LUZP2 was significantly related to LGG, KIRC, LUSC, and PRAD. Our results observed negative correlations of the stemness of LGG and PRAD with the mRNA expression of LUZP2, whose downregulation was closely associated with poor prognosis. The mutation frequencies of LGG, PRAD, KIRC, and LUSC were 0.4%, 0.4%, 0.3%, and 2.1%, respectively. We detected that the LUZP2 level was negatively associated with TILs in most cancers, including LGG, LUSC, PRAD, and KIRC, while the LUZP2 methylation showed the opposite results. In conclusion, the results of our initial pan-cancer investigation provided a somewhat thorough understanding of the functions of LUZP2 on KIRC, LGG, PRAD, and LUSC.

**Supplementary Information:**

The online version contains supplementary material available at 10.1186/s40164-022-00313-x.

## Dear Editor,

Age is a significant risk factor for many cancers, which can be exacerbated by global population ageing [1]. Cell damage caused by the passage of time is at the root of both cancer and aging [2]. Not only that, but aging is a strong predictor of the outcome of tumor treatment [3]. Leucine zipper protein 2 (LUZP2), a gene encoding leucine zipper protein 2, is located at 11p14.3 and is primarily expressed in brain tissue and prostate, which was rarely studied in the field of oncology before and its downregulation is associated with senescence (SeneQuest: http://Senequest.net) [4, 5].

In this study, we used oncological data from the Cancer Genome Atlas (TCGA) to perform a pan-cancer analysis of aging-associated gene LUZP2, mainly focusing on four types of cancers where LUZP2 is both differentially expressed between tumor and normal samples and prognostic-associated, including lower grade glioma (LGG), lung squamous cell carcinoma (LUSC), kidney renal clear cell carcinoma (KIRC) and prostate adenocarcinoma (PRAD) [6, 7]. The impact of tumor stemness, epigenetic regulation and tumor microenvironment (TME) on tumor aggressiveness and prognosis, as well as the underlying mechanism, were discussed. Our research has been submitted to the ISRCTN registry (No. ISRCTN11560295). We provided a full-text article in the Additional file 1 for the detailed methods and materials used in this study.

**Fig. 1:**
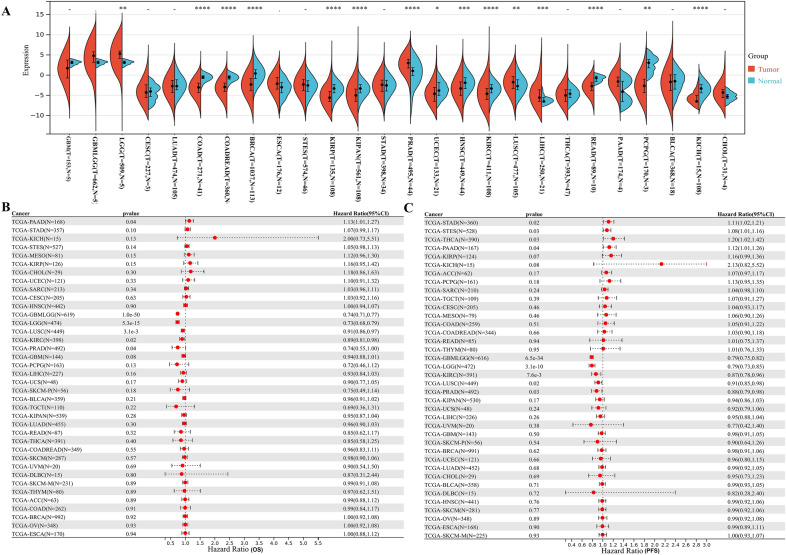
Differential expression and prognosis analysis of LUZP2. **A** Pan-cancer analysis of LUZP2 for differential expression between tumor and normal tissues; **B** Pan-cancer analysis of LUZP2 for OS; **C** Pan-cancer analysis of LUZP2 for PFS. *OS* overall survival, *PFS* progression-free survival

**Fig. 2:**
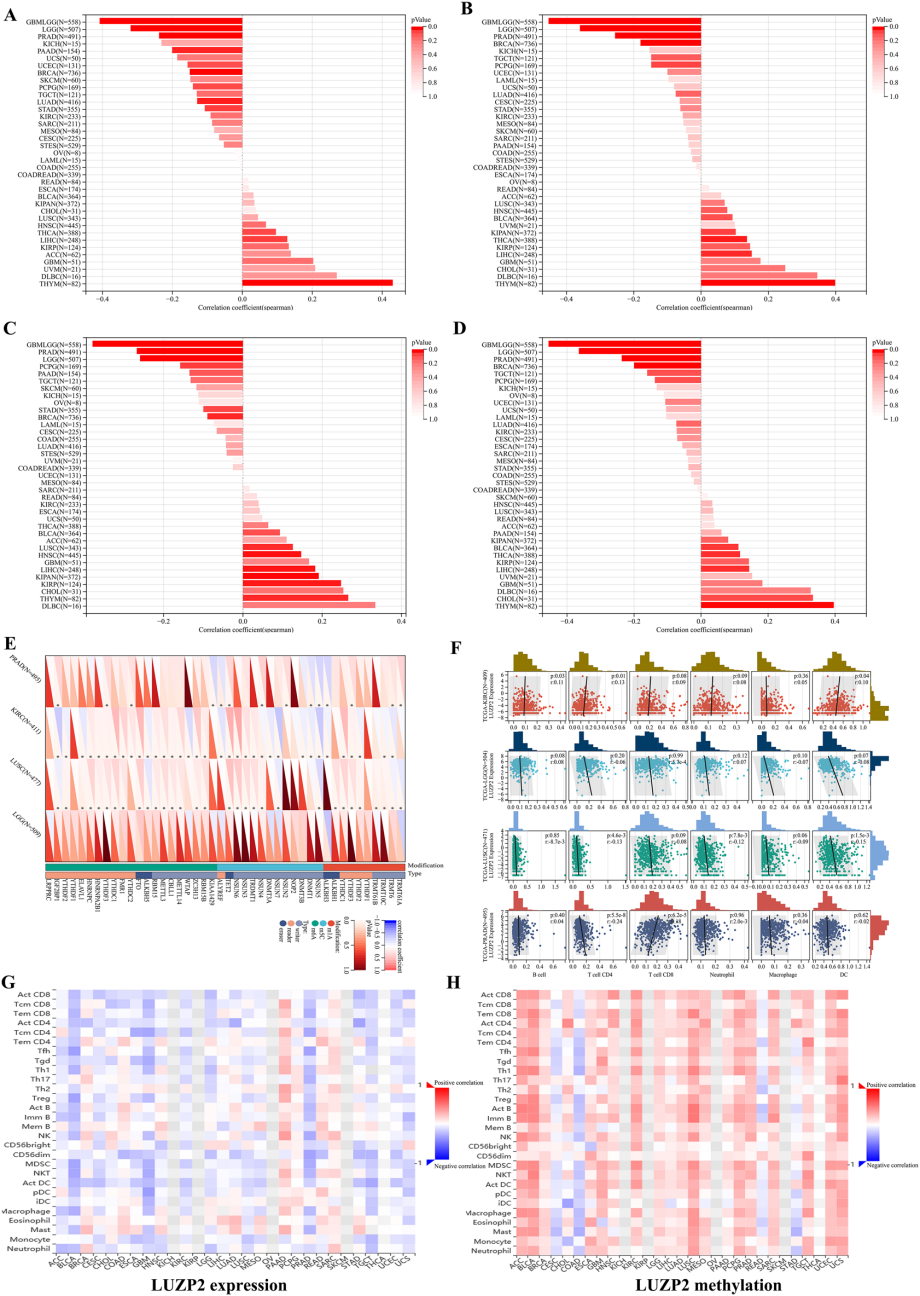
Results from the perspective of tumor stemness, epigenetic regulation and TME. **A** The correlation between tumor stemness and LUZP2 level using DMPss; **B** The correlation between tumor stemness and LUZP2 level using DNAss; **C** the correlation between tumor stemness and LUZP2 level using ENHss; **D** The correlation between tumor stemness and LUZP2 level using EREG-METHss; **E** The correlation of LUZP2 expression and RNA modification genes; **F** The correlation of LUZP2 expression with immune cells; **G** The correlation of LUZP2 expression with immune infiltrating cells in the TISIDB database; **H** The correlation of LUZP2 methylation with immune infiltrating cells in the TISIDB database. *TME* tumor microenvironment

In comparison to normal samples, we found that the LUZP2 mRNA expression was significantly higher in LGG, PRAD, LUSC and lower in KIRC and other eleven cancer species patients (Fig. 1A). In terms of overall survival, low-expression of LUZP2 was significantly associated with poor prognosis in LGG, PRAD, KIRC, and LUSC (Fig. 1B). Downregulation of LUZP2 was found to be significantly related to LGG, KIRC, LUSC, and PRAD in terms of progression-free survival (Fig. 1C). Recurrence, metastasis, drug resistance and poor prognosis are frequently associated with stemness [8]. Our findings revealed negative correlations between LGG and PRAD stemness and LUZP2 mRNA expression, which was associated with poor prognosis (Fig. 2A–D).

The m6As of certain mRNA in blood cells were found to be lower with age than in young human blood cells [9]. The methylation promoter region of mouse rRNA gradually and uniformly increases with age [10]. We hypothesized that RNA methylation was a link between aging and cancer. According to our findings, RNA methylation occurs most frequently in KIRC and LUSC, followed by PRAD, and LGG has almost no RNA methylation at the LUZP2 site (Fig. 2E). Given the importance of LUZP2 in aging, we hypothesized that epigenetic modification could affect LUZP2 expression or protein levels.

For patients with LGG, LUSC, and PRAD in our investigation, LUZP2 expression was significantly negatively connected with immune infiltration and positively correlated with tumor purity, with the exception of KIRC (Fig. 2F). In most malignancies, including LGG, LUSC, PRAD, and KIRC, we found that LUZP2 mRNA expression was inversely correlated with TILs (Fig. 2G), but LUZP2 methylation revealed the opposite results (Fig. 2H). We hypothesized that immunosenescence or tumor cell senescence was the underlying mechanism based on the observations mentioned above.

It is undeniable that the occurrence of most malignancies is increasing with age. However, it should be mentioned that the relationship between senescence and tumors is extremely convoluted from the microscopic level that causes aging and in terms of cell senescence. One potential explanation was that, in the early stages of tumor senescence, cell senescence hindered tumor growth, whereas in the late stages, it supported tumor progression [11]. Our initial examination of LUZP2 across all cancer types revealed statistical relationships between LUZP2 and tumor stemness, heterogeneity, immune infiltration, and clinical outcomes. We did, however, have to acknowledge that the majority of the study's conclusions called for more investigation.

## Conclusion

The results of our initial pan-cancer investigation provided a somewhat thorough understanding of the functions of LUZP2 on KIRC, LGG, PRAD, and LUSC.

## Supplementary Information

**Additional file 1: Figure S1**. The pan-cancer analysis of clinical correlation with LUZP2 expression. Differential expression and prognosis analysis of LUZP2. **Figure S2**. The pan-cancer Spearman analysis of tumor stemness and LUZP2 expression. **Figure S3**. The pan-cancer Spearman analysis of tumor heterogeneity and LUZP2 expression. **Figure S4**. Mutation landscapes analysis of LUZP2 and RNA modification. **Figure S5**. Tumor immune environment and its correlation with LUZP2 methylation

## Data Availability

The datasets presented in this study can be found in online repositories. The names of the repository/repositories and accession number(s) can be found in the article/Additional file 1.
