# Supplementary material for: A pan-cancer analysis of the oncogenic role of leucine zipper protein 2 in human cancer
Source: Exp Hematol Oncol. 2022 Sep 15;11:55. doi: 10.1186/s40164-022-00313-x (PMC9476580; doi:10.1186/s40164-022-00313-x)
Supplement: Supplementary file 1 — Additional file 1: Figure S1. The pan-cancer analysis of clinical correlation with LUZP2 expression. Differential expression and prognosis analysis of LUZP2. Figure S2. The pan-cancer Spearman analysis of tumor stemness and LUZP2 expression. Figure S3. The pan-cancer Spearman analysis of tumor heterogeneity and LUZP2 expression. Figure S4. Mutation landscapes analysis of LUZP2 and RNA modification. Figure S5. Tumor immune environment and its correlation with LUZP2 methylation [file 40164_2022_313_MOESM1_ESM.docx]

Supplementary figure 1. The pan-cancer analysis of clinical correlation with LUZP2 expression.


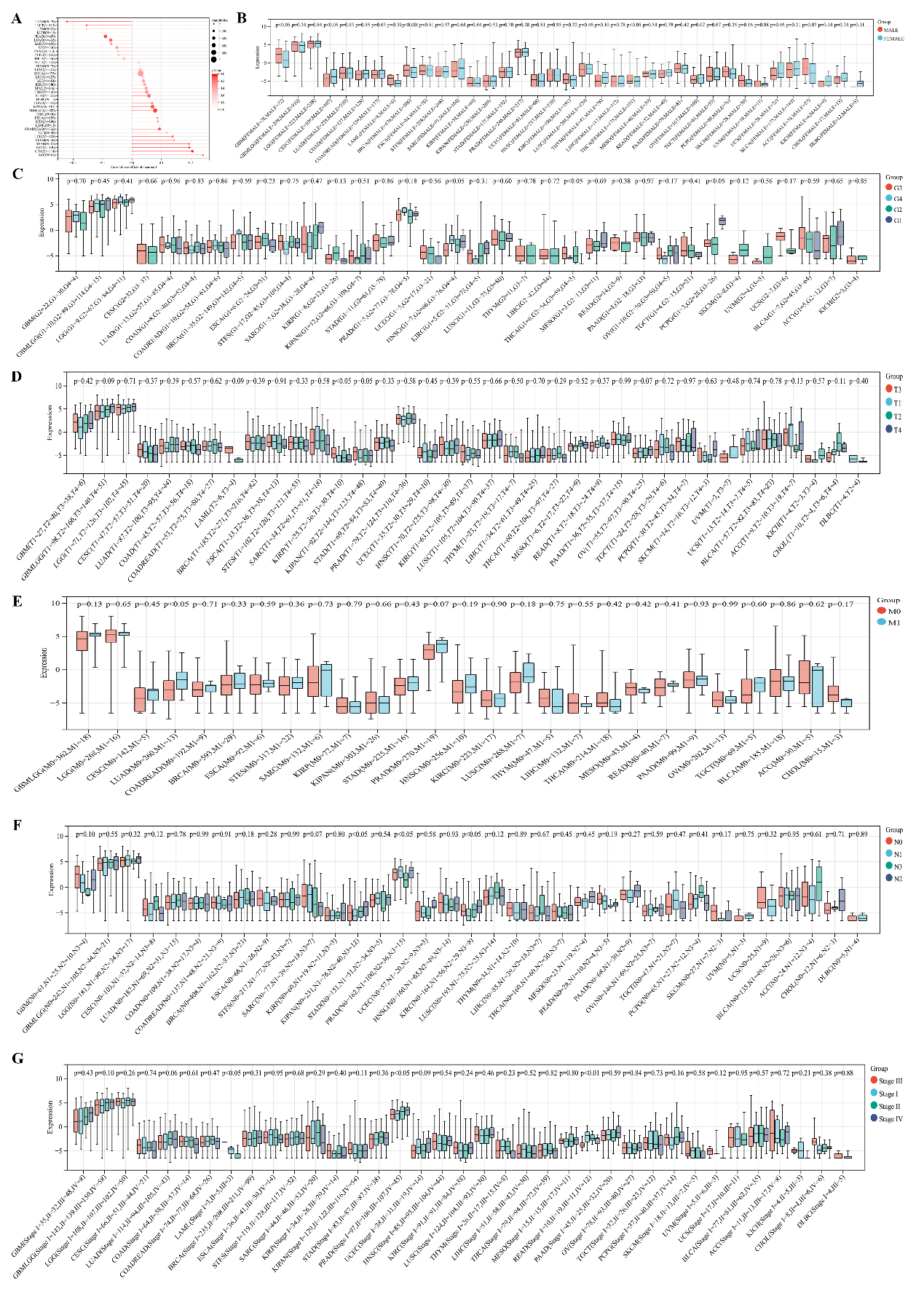


(A) the correlation of LUZP2 expression with age; (B) the correlation of LUZP2 expression with gender; (C) the correlation of LUZP2 expression with grade; (D) the correlation of LUZP2 expression with T stages; (E) the correlation of LUZP2 expression with M stage; (F) the correlation of LUZP2 expression with N stages; (G) the correlation of LUZP2 expression with clinical stages.

Supplementary figure 2. The pan-cancer Spearman analysis of LUZP2 expression and gene mutations.


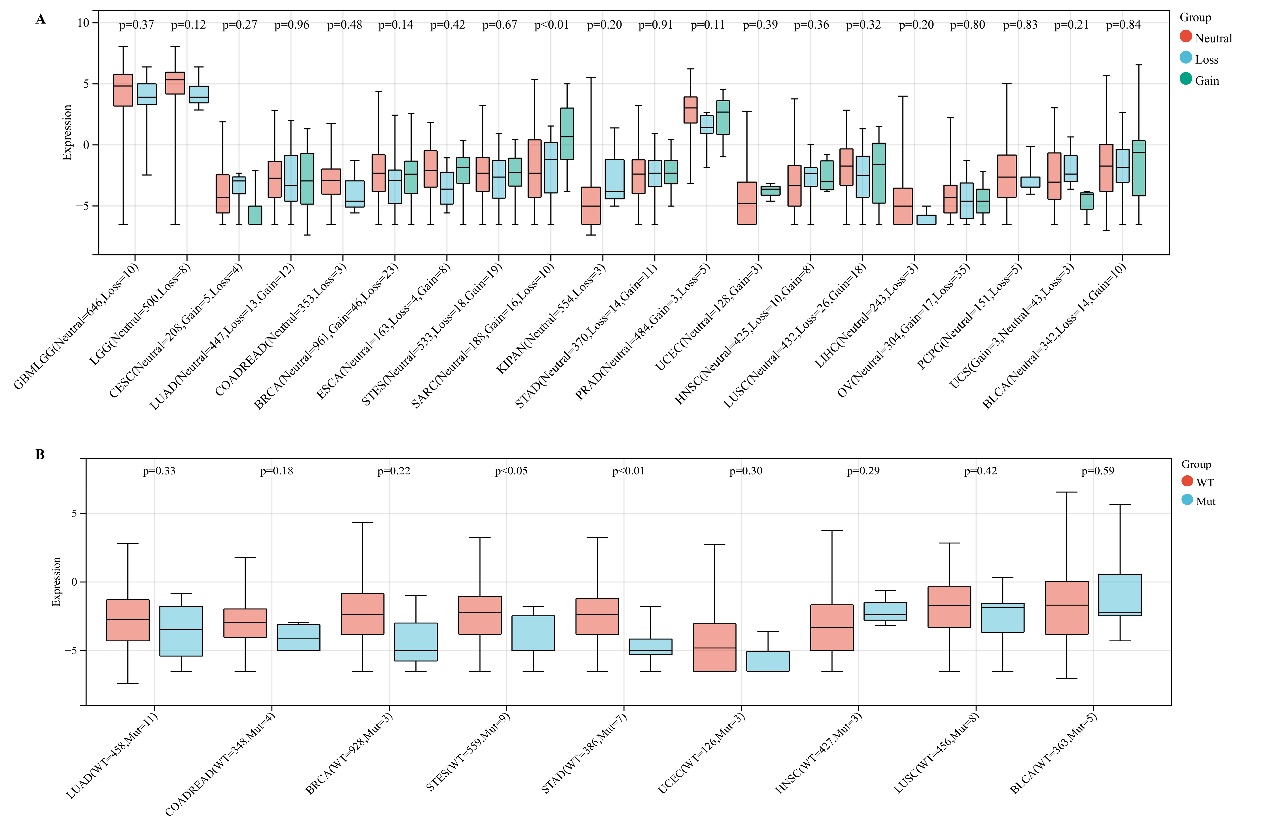


(A) the correlation of LUZP2 expression with simple nucleotide variation; (B) the correlation of LUZP2 expression with copy number variation.

**Abbreviations of cancers in the TCGA database**


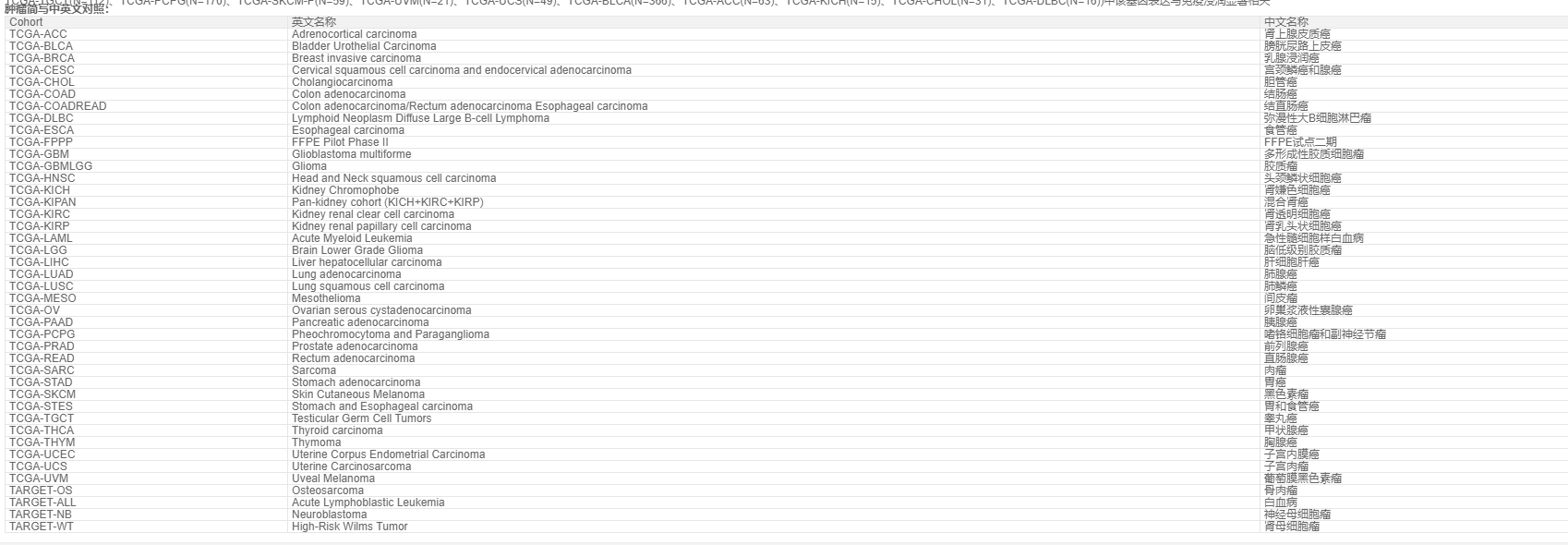


For readers to understand the detailed methods in the article, we provided the full-text article.

Title: Pan-cancer analysis of leucine zipper protein 2 with potential implications in prognosis and epigenetic modifications in cancers

**Running title:** The role of LUZP2 in cancers

**Authors:**

Dechao Feng, Ph.D.^1, *^, Xu Shi, M.D.^1, *^, Weizhen Zhu, M.D.^1, *^, Facai Zhang, Ph.D.^1^, Dengxiong Li, Ph.D.^1^, Wuran Wei, Ph.D.^1^, Ping Han, Ph.D.^1^, Qiang Wei, M.D.^1, &^, Lu Yang, Ph.D.^1, &^

^1^ Department of Urology, Institute of Urology, West China Hospital, Sichuan University, Chengdu 610041, China

^*^ These authors contributed equally to this work.

^&^ Corresponding Author: Department of Urology, Institute of Urology, West China Hospital, Sichuan University, Guoxue Xiang #37, Chengdu, 610041, Sichuan, People's Republic of China.

Tel: +86-28-85422444

Fax: +86-28-85422451

E-mail:

Qiang Wei: [weiqiang933@126.com](mailto:weiqiang933@126.com);

Lu Yang: [wycleflue@scu.edu.cn](mailto:wycleflue@scu.edu.cn).

**Abstract**

**Background:** To perform a pan-cancer analysis of leucine zipper protein 2 (LUZP2) from novel perspective of senescence.

**Methods:** A standardized TCGA pan-cancer dataset was downloaded. Differential expression, clinical prognosis, genetic mutations, immune infiltration, epigenetic modifications, tumor stemness and heterogeneity were analyzed. We conducted all analyses through software R 3.6.3 and its suitable packages.

**Results:** Overall, we found that the common cancers differentially expressed between tumor and normal samples and prognostic-associated were LGG, KIRC, LUSC, and PRAD in terms of OS or PFS. For PRAD patients, we found negative correlation of the LUZP2 with HRD, LOH, MSI, and ploidy. For KIRC, negative relationship between LUZP2 expression and LOH was found. In terms of LGG, we detected better negative correlation between LUZP2 expression and LOH and TMB, and positive correlation of LUZP2 expression with MATH. We found that the mRNA expression of LUZP2 was positively associated with LOH, tumor purity, NEO, and TMB for LUSC. The mutation frequencies of LGG, PRAD, KIRC, and LUSC were 0.4%, 0.4%, 0.3%, and 2.1%, respectively. Unlike the KIRC, LUZP2 expression was significantly negatively correlated with immune infiltration and positively correlates with tumor purity for LGG, LUSC, and PRAD patients. We detected that the LUZP2 level was negatively associated with TILs in most cancers, including LGG, LUSC, PRAD, and KIRC, while the LUZP2 methylation showed the opposite results.

**Conclusions:** Our first pan-cancer study offered a relatively comprehensive understanding of the roles of LUZP2 on KIRC, LGG, PRAD, and LUSC from the fresh perspective of senescence.

**Keywords:** tumor immune environment; pan-cancer analysis; RNA modification; DNA methylation; leucine zipper protein 2.

**Introduction**

With the increase in life expectancy, cancer has become the most important public health burden [1]. Cancer is a senile disease, and most cancers occur in people over 60 years of age. The basis of both cancer and aging are cell damage caused by the accumulation of time [2]. Not only that, aging serve also as a predictor for the outcome of tumor treatment to a large extent [3]. During the occurrence and development of cancer, cellular senescence, or senescence, is an inevitable concept. Senescence is defined as a permanent state of cell cycle arrest [4]. The cellular stress response of senescence is considered to be an effective tumor suppressor mechanism, while also a promotor of a series of proliferative and degenerative diseases, especially cancers [5].

Leucine zipper protein 2 (LUZP2), a gene encoding leucine zipper protein 2, is located at 11p14.3 and is mainly expressed in brain tissue and prostate, followed by breast, colon, lung, kidney and testis [6]. It was rarely studied in the field of oncology before. Previous studies have found that LUZP2 is also related to some non-neoplastic aging-related diseases such as Alzheimer's disease and other neurodegenerative and congenital diseases [7-9]. Li et al. discovered that LUZP2 was related to a poor prognosis of low-grade glioma (LGG), the underlying mechanism of which may be related to the development of the extracellular matrix (ECM) of the nervous system [10]. The expression of LUZP2 increased in the initial stages, but decreased when it gradually developed into high-grade gliomas [10]. Moreover, the expression of LUZP2 mRNA was found to increase in primary prostate cancer (PCa), but decreased in metastatic castration-resistant prostate cancer (mCPRC), which coincides with the results of glioma research, and silencing of LUZP2 inhibited the growth of enzalutamide-resistant C4-2 cells in vitro [11].

In this study, we drew the oncological data from the Cancer Genome Atlas (TCGA) to perform a pan-cancer analysis of aging-associated gene LUZP2 [12], mainly focusing on four types of cancers in which LUZP2 is both differentially expressed between tumor and normal samples and prognostic-associated, including LGG, lung squamous cell carcinoma (LUSC), renal clear cell carcinoma (KIRC) and prostate adenocarcinoma (PRAD). Our study has been registered in the ISRCTN registry (No. ISRCTN11560295).

**Methods**

**Differential and prognostic analysis**

We downloaded a standardized TCGA pan-cancer dataset from the UCSC database [13] and extracted the expression data of LUZP2 (ENSG00000187398) in each sample. We also screened the metastatic samples from primary blood derived cancer-peripheral blood (TCGA-LAML), primary tumor and TCGA-SKCM. In addition, we obtained a high-quality TCGA prognostic datasets from the previous TCGA prognostic study [14]. We filtered the samples with the expression level of 0 and the samples with the follow-up time shorter than 30 days, and further performed log2 (x+0.001) transformation for each expression value. We eliminated the cancer species with the number of samples less than 10, and finally obtained the expression data of 38 cancers and the data of overall survival (OS) and progression-free survival (PFS) of the corresponding samples. Cox proportional hazards regression model was used to analyze the prognostic value of LUZP2 on cancers, and log rank test was used to obtain prognostic significance. In terms of differential expression between tumor and normal samples, we screened the samples from sloid tissue normal, primary blood derived cancer-peripheral blood, primary tumor and removed the samples with the expression level of 0. Log2 (x+0.001) transformation for each expression value was performed as well, and cancers with the number of samples less than 3 were removed. Finally, we obtained the expression data of 26 cancers, and unpaired Wilcoxon rank sum and signed rank tests were used to perform differential significance analysis. The clinical correlations of LUZP2 in the pan cancer were evaluated as well. In this study, the abbreviations of each cancer from the TCGA database were shown in the supplementary material.

**Tumor stemness, heterogeneity, mutation landscape, and RNA modifications**

Four tumor stemness indexes, namely differentially methylated probes-based (DMPss), DNA methylation-based (DNAss), enhancer elements/DNA methylation-based (ENHss), epigenetically regulated DNA methylation-based (EREG-METHss), were used to analyze the correlation between stemness features and LUZP2 expression through the Spearman analysis [15]. In addition, homologous recombination deficiency (HRD) [16], loss of heterozygosity (LOH) [16], neoantigen (NEO) [16], tumor ploidy [16], tumor purity [16], mutant-allele tumor heterogeneity (MATH) and tumor mutation burden (TMB) obtained from the GDC (<https://portal.gdc.cancer.gov/>) and proceeded by MuTect2 software and R package “maftools” [17], and microsatellite instability (MSI) [18] were used to assess the relationship between tumor heterogeneity and LUZP2 expression. Simple nucleotide variation (SNV) and copy number variation (CNV) datasets of level 4 in the TCGA database were processed by MuTect2 [17] and GISTIC [19] software, respectively. We integrated the mutation data and gene expression data, and we filtered the synonymous mutation samples. In each investigated cancer, we assessed the difference in the frequency of gene mutations between high- and low-expression of LUZP2 according to the median expression of LUZP2 through the chi-square test.

**DNA methylation and tumor immune microenvironment (TME)**

We analyzed the correlations between LUZP2 and 44 marker genes of three types of RNA modification (10 of m1A, 13 of m5C, and 21 of m6A) through the Spearman analysis. The correlations of 24 inhibitory and 36 stimulatory checkpoints [16], and 150 immunoregulatory genes (chemokine (41), receptor (18), MHC (21), immunoinhibitory (24), immunostimulatory (46)) with the mRNA expression of LUZP2 were conducted as well. Timer [20] and ESTIMATE [21] algorithms were used to assess the TME using the R package “IOBR” [22]. In addition, we also analyzed the relationship between DNA methylation and mRNA expression of LUZP2, and the correlation of DNA methylation and mRNA expression of LUZP2 with tumor infiltrating lymphocytes (TILs) were performed through the TISIDB database [23].

**Statistical analysis**

We conducted all analyses through software R (version 3.6.3) and its suitable packages. Unpaired Wilcoxon rank sum and signed rank tests were used to analyze pairwise differences, and Kruskal test was used to test multiple sets of samples. Statistical significance was set as two-sided p < 0.05. Significance was marked as follows: *, p < 0.05; **, p < 0.01; ***, p < 0.001.

**Results**

**Differential and prognostic analysis**

Compared to normal samples, we observed that the LUZP2 mRNA expression was significantly upregulated in LGG, PRAD, LUSC, and liver hepatocellular carcinoma (LIHC) patients, and downregulated in colon adenocarcinoma (COAD), colorectal adenocarcinoma (COADREAD), breast invasive carcinoma (BRCA), kidney renal papillary cell carcinoma (KIRP), pan-kidney cohort (KIPAN), uterine corpus endometrial carcinoma (UCEC), head and neck squamous cell carcinoma (HNSC), kidney renal clear cell carcinoma (KIRC), rectum adenocarcinoma (READ), pheochromocytoma and paraganglioma (PCPG), and kidney chromophobe (KICH) patients (Fig. 1A). In terms of OS, we found that high-expression LUZP2 was significantly associated with poor prognosis in pancreatic adenocarcinoma (PAAD), and low-expression LUZP2 was significantly associated with poor prognosis in glioma (GBMLGG), LGG, PRAD, KIRC, and LUSC (Fig. 1B). For PFS, we observed that overexpression of LUZP2 was significantly related to stomach and esophageal carcinoma (STES), stomach adenocarcinoma (STAD), thyroid carcinoma (THCA), and PAAD, and downregulation of LUZP2 was significantly related to GBMLGG, LGG, KIRC, LUSC, and PRAD (Fig. 1C). Overall, we found that the common cancers differentially expressed between tumor and normal samples and prognostic-associated were LGG, KIRC, LUSC, and PRAD in terms of OS or PFS. Moreover, differential expression of LUZP2 was significant among N stages for PRAD and KIRC (supplementary fig. 1F), as well as for clinical stages of PRAD (supplementary fig. 1G).

**Tumor stemness, heterogeneity, mutation landscape, and RNA modifications**

The pan-cancer Spearman analysis showed that the mRNA expression of LUZP2 was negatively with four tumor stemness indexes (DMPss, DNAss, ENHss, and EREG-METHss) with statistical significance for LGG and PRAD (Fig. 2A-D). In terms of tumor heterogeneity, the mRNA expression of LUZP2 was significantly associated with TMB scores of the thymoma (THYM, r: 0.42), GBMLGG (r: -0.44), LGG (r: -0.28), lung adenocarcinoma (LUAD, r: -0.24), COAD (r: -0.28), COADREAD (r: -0.27), BRCA (r: -0.19), STES (r: -0.25), STAD (r: -0.29), and LUSC (r: -0.19) (Fig. 3A). LUZP2 expression was highly related to the MSI scores of the GBMLGG (r: 0.32), uveal melanoma (UVM, r: 0.45), cervical squamous cell carcinoma and endocervical adenocarcinoma (CESC, r: -0.15), COAD (r: -0.20), COADREAD (r: -0.17), STES (r: -0.16), KIPAN (r: -0.13), STAD (r: -0.24), and PRAD (r: -0.13) (Fig. 3B). LUZP2 expression was closely related to the HRD of the STES (r: 0.12), HNSC (r: 0.14), LUAD (r: -0.27), BRCA (r: -0.25), PRAD (r: -0.32), and LIHC (r: -0.17) (Fig. 3C). The mRNA expression of LUZP2 was significantly associated with LOH of the COAD (r: 0.15), COADREAD (r: 0.14), STES (r: 0.14), KIRP (r: 0.21), STAD (r=0.11), HNSC (r: 0.16), LUSC (r: 0.11), GBMLGG (r: -0.43), LGG (r: -0.30), LUAD (r: -0.17), BRCA (r: -0.26), PRAD (r: -0.15), KIRC (r: -0.19), and PCPG (r: -0.18) (Fig. 3D). The mRNA expression of LUZP2 was significantly associated with MATH of the GBMLGG (r: 0.36), LGG (r: 0.24), COAD (r: 0.13), COADREAD (r: 0.13), STES (r: 0.11), THYM (r: 0.23), BRCA (r: -0.09), UCEC (r: -0.21), and LIHC (r: -0.16) (Fig. 3E). The mRNA expression of LUZP2 was significantly associated with NEO of the LUAD (r: -0.22), COAD (r: -0.21), COADREAD (r: -0.25), BRCA (r: -0.12), LUSC (r: -0.12), and READ (r: -0.29) (Fig. 3F). In addition, the mRNA expression of LUZP2 was significantly associated with tumor ploidy of the 12 cancers, such as LGG (r: 0.10) and PRAD (r: -0.13) (Fig. 3G). The mRNA expression of LUZP2 was significantly associated with tumor purity of 16 cancers, including LGG (r: 0.09) and LUSC (r: 0.15) (Fig. 3H).

The mutation frequencies of LGG, PRAD, KIRC, and LUSC were 0.4%, 0.4%, 0.3%, and 2.1%, respectively (Fig. 4A). We divided tumor patients into two groups according to the median expression of LUZP2. The mutations of TACC2, NPHP3, and MAGEC1 were significant between high- and low-expression of LUZP2 for KIRC (Fig. 4B). In terms of LGG, the top 15 genetic mutations were significant between high- and low-expression of LUZP2, including IDH1, TP53, ATRX, CIC, EGFR, NF1, PTEN, SMARCA4, KAT6B, RYR1, ROS1, SI, RB1, A2M, and GIGYF2 (Fig. 4C). TP53, KMT2D, EPB41L3, CASZ1, PTPRD, MYH10, and CHD5 mutations were significant for PRAD (Fig. 4D), and PAPPA2, PCDH15, HCN1, NFE2L2, APOB, FAT1, DNAH9, ANK2, MDGA2, MYO18B, PDZD2, NLRP3, SPEF2, SPATA31D1, and TRPS1 mutations were significant for LUSC (Fig. 4E) between high- and low-expression of LUZP2. Besides, the correlation of the LUZP2 expression with SNV and CNV was not obvious for LGG, KIRC, LUSC, or PRAD (supplementary fig. 2). In terms of PRAD, the mRNA expression of LUZP2 was positively associated with TRMT61A, TRMT6, YTHDF3, YTHDC2, NSUN2, NSUN4, TET2, HNRNPA2B1, and IGF2BP1, while negatively associated with ALKBH3, NSUN5, DNMT1, ALYREF, and KIAA1429 (Fig. 4F). The LUZP2 mRNA expression was closely related to most marker genes of RNA modifications for KIRC and LUSC, where the mRNA expression of LUZP2 was mainly related to most marker genes of RNA modifications with positive correlation for LUSC (Fig. 4F). No significant correlation of the LUZP2 expression with RNA modification genes was observed for LGG (Fig. 4F).

**DNA methylation and TME**

We observed that the LUZP2 mRNA expression was positively associated with B cells, CD4+ T cells, dendritic cells (DCs), and stromal score with statistical significance for KIRC patients (Fig. 5A-D). In contrast, the LUZP2 mRNA expression was negatively associated with CD4+ T cells, neutrophils, DCs, stromal score, immune score, and estimate score for LUSC patients (Fig. 5A-D). Similarly, we found significantly negative correlations of the LUZP2 mRNA expression with CD4+ T cells, CD8+ T cells, stromal score, immune score, and estimate score for PRAD patients (Fig. 5A-D). No significant relationship between tumor infiltrating cells and the LUZP2 mRNA expression in terms of the LGG patients, but the LUZP2 mRNA expression was negatively related to the stromal score with statistical significance (Fig. 5A-D). We detected that the mRNA expression of LUZP2 was negatively associated with TILs in most cancers, including LGG, LUSC, PRAD, and KIRC (Fig. 5E), while the LUZP2 methylation showed the opposite results (Fig. 5F). The Spearman analysis showed that the mRNA expression of LUZP2 was significantly associated with most immune checkpoints for the LGG, PRAD, and LUSC patients, and no significant correlation of the LUZP2 mRNA expression with immune checkpoints was observed (Fig. 6A). Besides, we found that the LUZP2 mRNA expression was negatively related to the immune regulatory genes to a large extent for KIRC, PRAD, LUSC, and LGG patients (Fig. 6B).

**Discussion**

Leucine zipper structures often appear at the C-terminus of eukaryotic DNA binding proteins, such as yeast transcription activator GCN4, oncoprotein Jun, Fos, Myc, etc., which are often related to oncogene expression and regulation functions [24]. LUZP2 can encode leucine zipper protein 2 and is an aging-related gene, whose downregulation is associated with senescence [6, 12]. Aging is considered as a part of the phenotype of cancer. For example, aging-related genes are overexpressed in high-grade gliomas and indicate poor prognosis [25-27]. Moreover, senescent cells produce and secrete a large number of senescence-associated secretory phenotype (SASP), such as hyaluronic acid in brain, which can support single cell invasion and NF-κB activation, as well as fibronectin, which is a component of the ECM to promote metastasis and invasiveness [28, 29]. IL-6 secreted by senescent cells in the glioma microenvironment is an independent indicator to age, grade, and prognosis, and it activates the cytokine network STAT3, which is related to inflammation and angiogenesis [25]. Here, we confirmed that LUZP2 was differentially expressed between four cancer types with normal tissues. Similar to the previous studies on LGG and PRAD [10-11], we found that LUZP2 downregulation contributed to worse prognosis of LGG and PRAD in this study. Moreover, patients with low-expression LUZP2 had shorter OS and PFS than those with high-expression LUZP2 as well. Furthermore, from the perspective of tumor stemness, heterogeneity, mutation, TME, and epigenetic regulation, the impact of these factors on tumor aggressiveness and prognosis and the underlying mechanism were discussed.

In our study, four methods calculated by mRNA expression and methylation signature were used to measure the stemness size of four cancers [15]. The stemness of a tumor is defined as the potential for self-renewal and differentiation from the cell-of-origin during the loss of the differentiated phenotype and the acquisition of stem cell-like characteristics [30]. Stemness is often related to recurrence, metastasis, drug resistance and poor prognosis [31]. Similarly, our results observed negative correlations of the stemness of LGG and PRAD with the mRNA expression of LUZP2, whose downregulation was closely associated with poor prognosis. Given the role of LUZP2 on senescence, we proposed that epigenetic modification might involve in the expression or protein level of LUZP2. From the perspective of LGG, this result may be due to the high frequency of IDH1/2 mutations and the resulting DNA hypermethylation, which will be the focus of discussion in our following paragraph [32, 33]. Glioma stem cells (GSC) with tumor-initiating characteristics has been identified and separated in malignant gliomas [34, 35]. In addition, the Notch signaling pathway, Sonic Hedgehog, Wnt/β-catenin, Akt and STAT3 signaling pathways are related to the maintenance of LGG stemness [36]. This GSC has the potential to induce angiogenesis, metastasis and modulate therapeutic response [37]. Compared with LGG, there is no uniform definition of cancer stem-like cells (CSCs) in PRAD. Zhang et al. established intron retention caused by splicing regulatory gene dysregulation as a sign of PRAD's stemness and invasiveness [38]. In addition, CSC in PRAD is also defined as aggressive and treatment-resistant tumor cells that are not affected by standard androgen receptor targeted therapies [39]. In conclusion, since senescence can fight against the self-reproduction ability of stem cells and is related to the gradual depletion of the stem cell pool, aging-related genes including LUZP2 may be potential targets for these cancer treatments [40].

In terms of PRAD patients, we found negative correlation of the LUZP2 with HRD, LOH, MSI, and ploidy, among which the correlation between HRD and LUZP2 mRNA expression was higher than others. DNA nonploidy was independently associated with reduced OS for PRAD patients [41], which was consistent with our result. However, other studies reported the LUZP2 expression decreased in mCRPC [11] and HRD was very common in PRAD, especially metastatic prostate cancer, suggesting that HRD targeted therapy by PARPi, the currently approved treatment for breast and ovarian cancer, may be beneficial to PRAD [42, 43]. Thus, we thought the epigenetic regulation of LUZP2 occupied a dominant position in the PRAD. LOH is a chromosomal event that can cause the loss of the entire gene and the nearby chromosomal region [16]. For KIRC, negative relationship between LUZP2 expression and LOH was found. Mitsumori et al. found that the LOH ratio was significantly related to tumor size, AgNOR score, and histological grade, but not an independent predictor of survival after adjusting [44]. Importantly, the loss of heterozygosity on chromosome 3p was associated with telomerase activity in KIRC. These genes on chromosome 3p could encode proteins that were important for aging and/or immortalization, further suggesting the relationship between aging and KIRC potential relationship [45]. In terms of LGG, we detected better negative correlation between LUZP2 expression and LOH and TMB, and positive correlation of LUZP2 expression with MATH. Consistent with our results, the previous meta-analysis showed that codeletion of 1p and 19q was associated with a better PFS and OS for LGG [46]. Intra-tumor heterogeneity is one of the most important causes of therapy resistance, which eventually leads to the poor outcomes observed in patients with glioma [47]. MATH could precisely reflect genetic intra-tumor heterogeneity and MATH values were negatively associated with the 2- and 5-year recurrence-free survival rates in patients with glioma [47], supporting the opinion that epigenetic regulation of LUZP2 played an important role in LGG. Besides, previous study found that higher TMB was highly associated with improved survival in patients with bladder cancer receiving immune checkpoint inhibitor treatments [47]. Thus, patients with high-expression LUZP2 might be suitable for immune checkpoint inhibitors when considering the effect of epigenetic regulation on LUZP2 level. LOH involving several chromosome 3p regions has been detected in almost 100% of small cell lung cancers and more than 90% of non-small cell lung cancers, accompanied by chromosome 3p deletions [49]. Moreover, LOH increases with the continuous accumulation of molecular barriers in cells and the degree of tumor progression [50]. For untreated non-small cell lung cancers, immune-infiltrated tumor regions exhibited ongoing immunoediting, with either LOH in human leukocyte antigens or depletion of expressed NEO, and promoter hypermethylation of genes containing neoantigenic mutations could serve as an epigenetic mechanism of immunoediting [51]. In this study, we found that the mRNA expression of LUZP2 was positively associated with LOH, tumor purity, NEO, and TMB. Similar to the above conjectures of LGG and PRAD, we thought epigenetic regulation of LUZP2 expression might exist in the LUSC patients.

A total of 90% of the main events in KIRC are biallelic loss of chromosome 3p and the resulting four tumor suppressor genes, including VHL, PBRM1, BAP1, and SETD2 [52]. The loss of VHL leads to the overdrive of HIF1/2 signal and the accompanying excessive formation of tumor blood vessels is the principle of using vascular endothelial growth factor receptor (VEGFR) inhibitors [52]. About 80% of LGG tumors carry IDH1/2 mutations, which confers a genome-wide hypermethylator phenotype [33]. The CpG island methylation phenotype established by this single-gene mutation is a powerful determinant of tumor pathogenicity [32, 53]. PAPPA2 encodes an enzyme that regulates insulin-like growth factor-I (IGF-I) and is associated with increased mortality in lung cancer patients [54]. Its main point of action is concentrated in growth physiology, and there are few studies in LUSC, which may be a future direction [55]. TP53 mutations are common in lethal primary PCa and RB1/TP53/AR aberrations are enriched in later stages [56, 57]. In addition, the loss of TP53 and RB1 function is related to the development of resistance to the antiandrogen drug enzalutamide, through the mechanism of lineage plasticity to allow PCa cells to escape from targeted therapy [58]. Genome-wide genetic and epigenetic inheritance together guide the development of cancer. More than 70 types of RNA methylation have been identified so far, among which m6A is the most abundant modification detected and the best characterization at the functional level. With aging, the m6As of certain mRNA in blood cells was found to be less than that in young human blood cells [59]. And the methylation promoter region of mouse rRNA increases gradually and universally with age [60]. We speculated that RNA methylation may be the link between aging and cancer. Our results show that RNA methylation occurs most often in KIRC and LUSC, followed by PRAD, and LGG has almost no RNA methylation in LUZP2 site. For KIRC, according to the m6A RNA methylation regulator, three subgroups were identified. Among them, the KIRC2 subgroup has the lowest tumor mutation burden level and the highest co-inhibitory molecule expression level, and its poor prognosis is considered to be mediated by immune evasion [61]. In addition, epigenetic drift is also one of the driving forces that induce the formation of an aged transcriptome, which will be what we will focus on in the next paragraph [62, 63]. For LUSC, consistent with our results, Feng et al. found that the m6A RNA methylation regulatory gene expression level in the high-risk prognosis group was lower [64]. Moreover, m5C modulators can predict clinical prognostic risk and regulate the tumor immune microenvironment in LUSC [65]. Compared with tumor-free mice, the total m6A RNA methylation level of PRAD mice was significantly increased, which was also related to Gleason grade [66]. In addition, although our study did not find a significant connection, different literatures have stated that RNA methylation, especially M6A in glioma cell apoptosis and tumor biogenesis, migration and invasion [67, 68].

Aging is accompanied by changes in the immune system, namely immunosenescence, manifested as the decline of immune function, the decrease in the development of lymphocytes, and the accumulation of inflammatory mediators derived from SASP [69-71]. SASP transforms senescent fibroblasts into pro-inflammatory cells with the ability to promote tumor progression, thereby realizing a positive impact on tumor progression [72]. Stromal cells and immune cells in TME are believed to have essential role in promoting or inhibiting anti-cancer immunity [73]. Although the TMEs of tumors originating from different organs and tumors originating from the same organ or tissue are significantly different, the results of our analysis are consistent, except for KIRC. Unlike the KIRC, LUZP2 expression was significantly negatively correlated with immune infiltration and positively correlates with tumor purity for LGG, LUSC, and PRAD patients in our study. Combined with the above findings, we speculated that immunosenescence or tumor cell senescence were the underlying mechanism. For KIRC patients, LUZP2 was downregulated compared to normal samples, and the correlation of LUZP2 level with tumor heterogeneity and stemness were not apparent. Thus, we thought downregulation of LUZP2 contributed to the immunosenescence or stromal senescence, and further contributed to the poor prognosis of KIRC. This hypothesis was supported by the results that LUZP2 level was primarily and negatively associated with immune regulatory genes for KIRC, PRAD, and LUSC. In contrast, low-expression LUZP2 promoted the senescence of TME and tumor cells, and the senescence of tumor cells were dominant, which were partially supported by the previous study showing that silencing of LUZP2 inhibited the growth of enzalutamide-resistant C4-2 cells [11]. However, the LUZP2 mRNA expression was higher in these samples compared to the corresponding normal tissues, and high-expression LUZP2 was closely associated with better prognosis. Combined with the opposite effects of LUZP2 expression and methylation on TILs in this study, we thought epigenetic modifications, including RNA modifications and DNA methylation, decreased the expression or protein level of LUZP2 in these tumor patients, which could be partially supported by the decreased LUZP2 level in mCRPC [11].

There is no doubt that with age, the prevalence of most tumors is showing an upward trend. However, from the microscopic level that causes aging, and in terms of cell senescence, it should be noted that the relationship between senescence and tumors is very complicated. One possible mechanism was that cell senescence inhibited tumor growth in the early stage of tumor senescence, and promoted tumor progression in the late stage of tumor senescence [74]. This view was consistent with previous studies on the expression level of LUZP2 in prostate cancer and glioma [10, 11]. LUZP2 should not be classified as an oncogene in the traditional sense. It seemed that LUZP2 might interact with other genes to influence the DNA methylation level of tumor cells and ECM components by promoting cell senescence, thus inhibited and promoted cancer in the early stage of occurrence and the late stage of metastasis and invasion, respectively. Taken together, our first pan-cancer analysis of LUZP2 indicated statistical correlation of LUZP2 with clinical prognosis, immune infiltration, epigenetic modifications, tumor stemness and heterogeneity. However, we had to admit that most of findings in this study, including the proposed mechanism, warranted to be further studied.

**Conclusion**

Our first pan-cancer study offered a relatively comprehensive understanding of the roles of LUZP2 on KIRC, LGG, PRAD, and LUSC from the fresh perspective of senescence.

**Declarations**

**Ethical Approval and Consent to participate**

The authors are accountable for all aspects of the work in ensuring that questions related to the accuracy or integrity of any part of the work are appropriately investigated and resolved.

**Consent for publication**

Not applicable.

**Availability of supporting data**

The datasets presented in this study can be found in online repositories. The names of the repository/repositories and accession number(s) can be found in the article/supplementary material.

**Competing interests**

The authors have no conflicts of interest to declare.

**Funding**

This program was supported by the National Natural Science Foundation of China (Grant Nos. 81974099, 82170785, 81974098, 82170784), programs from Science and Technology Department of Sichuan Province (Grant Nos. 21GJHZ0246), Young Investigator Award of Sichuan University 2017 (Grant No. 2017SCU04A17), Technology Innovation Research and Development Project of Chengdu Science and Technology Bureau (2019-YF05-00296-SN), Sichuan University--Panzhihua science and technology cooperation special fund (2020CDPZH-4). The funders had no role in study design, data collection or analysis, preparation of the manuscript, or the decision to publish.

**Authors' contributions**

DCF, XS, and WZZ proposed the project, conducted data analysis, interpreted the data, and wrote the manuscript; FCZ, DXL, and PH conducted data analysis, interpreted the data; QW and LY, supervised the project, and interpreted the data; All authors reviewed and edited the manuscript.

**Acknowledgements**

The results showed here are in whole or part based upon data generated by the TCGA Research Network: <https://www.cancer.gov/tcga>.

**References**

1. Siegel RL, Miller KD, Fuchs HE, et al. Cancer Statistics, 2021. CA Cancer J Clin. 2021;71(1):7-33.

2. Aunan JR, Cho WC, Soreide K. The Biology of Aging and Cancer: A Brief Overview of Shared and Divergent Molecular Hallmarks. Aging Dis. 2017;8(5):628-42.

3. Fane M, Weeraratna AT. How the ageing microenvironment influences tumour progression. Nat Rev Cancer. 2020;20(2):89-106.

4. Hayflick L, Moorhead PS. The serial cultivation of human diploid cell strains. Exp Cell Res. 1961; 25:585-621.

5. Campisi J. Aging, cellular senescence, and cancer. Annu Rev Physiol. 2013; 75:685-705.

6. Szabo L, Morey R, Palpant NJ, et al. Statistically based splicing detection reveals neural enrichment and tissue-specific induction of circular RNA during human fetal development. Genome Biol. 2015; 16:126.

7. Stepanov V, Vagaitseva K, Bocharova A, et al. Analysis of Association of Genetic Markers in the LUZP2 and FBXO40 Genes with the Normal Variability in Cognitive Performance in the Elderly. Int J Alzheimers Dis. 2018; 2018:2686045.

8. Cummings AC, Jiang L, Velez Edwards DR, et al. Genome-wide association and linkage study in the Amish detects a novel candidate late-onset Alzheimer disease gene. Ann Hum Genet. 2012;76(5):342-51.

9. Wu M, Michaud EJ, Johnson DK. Cloning, functional study and comparative mapping of Luzp2 to mouse chromosome 7 and human chromosome 11p13-11p14. Mamm Genome. 2003;14(5):323-34.

10. Li Y, Deng G, Qi Y, et al. Downregulation of LUZP2 Is Correlated with Poor Prognosis of Low-Grade Glioma. Biomed Res Int. 2020;2020:9716720.

11. Zhao J, Zhao Y, Wang L, et al. Alterations of androgen receptor-regulated enhancer RNAs (eRNAs) contribute to enzalutamide resistance in castration-resistant prostate cancer. Oncotarget. 2016;7(25):38551-65.

12. Gorgoulis V, Adams PD, Alimonti A, et al. Cellular Senescence: Defining a Path Forward. Cell. 2019;179(4):813-827.

13. Goldman MJ, Craft B, Hastie M, et al. Visualizing and interpreting cancer genomics data via the Xena platform. Nat Biotechnol. 2020;38(6):675-678.

14. Liu J, Lichtenberg T, Hoadley KA, et al. An Integrated TCGA Pan-Cancer Clinical Data Resource to Drive High-Quality Survival Outcome Analytics. Cell. 2018;173(2):400-416.e11.

15. Malta TM, Sokolov A, Gentles AJ, et al. Machine Learning Identifies Stemness Features Associated with Oncogenic Dedifferentiation. Cell. 2018;173(2):338-354.e15.

16. Thorsson V, Gibbs DL, Brown SD, et al. The Immune Landscape of Cancer. Immunity. 2018;48(4):812-830.e14.

17. Beroukhim R, Mermel CH, Porter D, et al. The landscape of somatic copy-number alteration across human cancers. Nature. 2010;463(7283):899-905.

18. Bonneville R, Krook MA, Kautto EA, et al. Landscape of Microsatellite Instability Across 39 Cancer Types. JCO Precis Oncol. 2017;2017:PO.17.00073.

19. Mermel CH, Schumacher SE, Hill B, et al. GISTIC2.0 facilitates sensitive and confident localization of the targets of focal somatic copy-number alteration in human cancers. Genome Biol. 2011;12(4): R41.

20. Li T, Fan J, Wang B, et al. TIMER: A Web Server for Comprehensive Analysis of Tumor-Infiltrating Immune Cells. Cancer Res. 2017;77(21): e108-e110.

21. Yoshihara K, Shahmoradgoli M, Martínez E, et al. Inferring tumour purity and stromal and immune cell admixture from expression data. Nat Commun. 2013;4:2612.

22. Zeng D, Ye Z, Shen R, et al. IOBR: Multi-Omics Immuno-Oncology Biological Research to Decode Tumor Microenvironment and Signatures. Front Immunol. 2021; 12:687975.

23. Ru B, Wong CN, Tong Y, et al. TISIDB: an integrated repository portal for tumor-immune system interactions. Bioinformatics. 2019;35(20):4200-4202.

24. Alber T. Structure of the leucine zipper. Curr Opin Genet Dev. 1992;2(2):205-10.

25. Coppola D, Balducci L, Chen DT, et al. Senescence-associated-gene signature identifies genes linked to age, prognosis, and progression of human gliomas. J Geriatr Oncol. 2014;5(4):389-99.

26. Yang Y, Chen X, Sun J, et al. Cell aging related genes can be used to characterize clinical prognoses and further stratify diffuse gliomas. Sci Rep. 2021;11(1):19493.

27. Xiao G, Zhang X, Zhang X, et al. Aging-related genes are potential prognostic biomarkers for patients with gliomas. Aging (Albany NY). 2021;13(9):13239-63.

28. Cargill R, Kohama SG, Struve J, et al. Astrocytes in aged nonhuman primate brain gray matter synthesize excess hyaluronan. Neurobiol Aging. 2012;33(4):830 e13-24.

29. Hernandez-Segura A, de Jong TV, Melov S, et al. Unmasking Transcriptional Heterogeneity in Senescent Cells. Curr Biol. 2017;27(17):2652-60 e4

30. Friedmann-Morvinski D, Verma IM. Dedifferentiation and reprogramming: origins of cancer stem cells. EMBO Rep. 2014;15(3):244-53.

31. Shibue T, Weinberg RA. EMT, CSCs, and drug resistance: the mechanistic link and clinical implications. Nat Rev Clin Oncol. 2017;14(10):611-29.

32. Noushmehr H, Weisenberger DJ, Diefes K, et al. Identification of a CpG island methylator phenotype that defines a distinct subgroup of glioma. Cancer Cell. 2010;17(5):510-22.

33. Turcan S, Rohle D, Goenka A, et al. IDH1 mutation is sufficient to establish the glioma hypermethylator phenotype. Nature. 2012;483(7390):479-83.

34. Galli R, Binda E, Orfanelli U, et al. Isolation and characterization of tumorigenic, stem-like neural precursors from human glioblastoma. Cancer Res. 2004;64(19):7011-21.

35. Singh SK, Hawkins C, Clarke ID, et al. Identification of human brain tumour initiating cells. Nature. 2004;432(7015):396-401.

36. Yi L, Zhou X, Li T, et al. Notch1 signaling pathway promotes invasion, self-renewal and growth of glioma initiating cells via modulating chemokine system CXCL12/CXCR4. J Exp Clin Cancer Res. 2019;38(1):339.

37. Huang Z, Cheng L, Guryanova OA, et al. Cancer stem cells in glioblastoma--molecular signaling and therapeutic targeting. Protein Cell. 2010;1(7):638-55.

38. Zhang D, Hu Q, Liu X, et al. Intron retention is a hallmark and spliceosome represents a therapeutic vulnerability in aggressive prostate cancer. Nat Commun. 2020;11(1):2089.

39. Laudato S, Aparicio A, Giancotti FG. Clonal Evolution and Epithelial Plasticity in the Emergence of AR-Independent Prostate Carcinoma. Trends Cancer. 2019;5(7):440-55.

40. Garcia-Prat L, Martinez-Vicente M, Perdiguero E, et al. Autophagy maintains stemness by preventing senescence. Nature. 2016;529(7584):37-42

41. Pollack A, Grignon DJ, Heydon KH, et al. Prostate cancer DNA ploidy and response to salvage hormone therapy after radiotherapy with or without short-term total androgen blockade: an analysis of RTOG 8610. J Clin Oncol. 2003;21(7):1238-48.

42. Nguyen L, J WMM, Van Hoeck A, et al. Pan-cancer landscape of homologous recombination deficiency. Nat Commun. 2020;11(1):5584.

43. Hoppe MM, Sundar R, Tan DSP, et al. Biomarkers for Homologous Recombination Deficiency in Cancer. J Natl Cancer Inst. 2018;110(7):704-13.

44. Mitsumori K, Kittleson JM, Itoh N, et al. Chromosome 14q LOH in localized clear cell renal cell carcinoma. J Pathol. 2002;198(1):110-4.

45. Mehle C, Lindblom A, Ljungberg B, et al. Loss of heterozygosity at chromosome 3p correlates with telomerase activity in renal cell carcinoma. Int J Oncol. 1998;13(2):289-95.

46. Zhao J, Ma W, Zhao H. Loss of heterozygosity 1p/19q and survival in glioma: a meta-analysis. Neuro Oncol. 2014;16(1):103-12.

47. Wu P, Yang W, Ma J, et al. Mutant-allele tumor heterogeneity in malignant glioma effectively predicts neoplastic recurrence. Oncol Lett. 2019;18(6):6108-6116.

48. Samstein RM, Lee CH, Shoushtari AN, et al. Tumor mutational load predicts survival after immunotherapy across multiple cancer types. Nat Genet. 2019;51(2):202-206.

49. Zabarovsky ER, Lerman MI, Minna JD. Tumor suppressor genes on chromosome 3p involved in the pathogenesis of lung and other cancers. Oncogene. 2002;21(45):6915-35.

50. Shen C, Wang X, Tian L, et al. Microsatellite alteration in multiple primary lung cancer. J Thorac Dis. 2014;6(10):1499-505.

51. Rosenthal R, Cadieux EL, Salgado R, et al. Neoantigen-directed immune escape in lung cancer evolution. Nature. 2019;567(7749):479-485.

52. Hsieh JJ, Le VH, Oyama T, et al. Chromosome 3p Loss-Orchestrated VHL, HIF, and Epigenetic Deregulation in Clear Cell Renal Cell Carcinoma. J Clin Oncol. 2018: JCO2018792549.

53. Jones PA, Baylin SB. The epigenomics of cancer. Cell. 2007;128(4):683-92.

54. Hjortebjerg R, Espelund U, Rasmussen TR, et al. Pregnancy-Associated Plasma Protein-A2 Is Associated With Mortality in Patients With Lung Cancer. Front Endocrinol (Lausanne). 2020;11:614.

55. Fujimoto M, Andrew M, Dauber A. Disorders caused by genetic defects associated with GH-dependent genes: PAPPA2 defects. Mol Cell Endocrinol. 2020;518:110967.

56. Mateo J, Seed G, Bertan C, et al. Genomics of lethal prostate cancer at diagnosis and castration resistance. J Clin Invest. 2020;130(4):1743-51.

57. Quigley DA, Dang HX, Zhao SG, et al. Genomic Hallmarks and Structural Variation in Metastatic Prostate Cancer. Cell. 2018;174(3):758-69 e9.

58. Mu P, Zhang Z, Benelli M, et al. SOX2 promotes lineage plasticity and antiandrogen resistance in TP53- and RB1-deficient prostate cancer. Science. 2017;355(6320):84-8.

59. Min KW, Zealy RW, Davila S, et al. Profiling of m6A RNA modifications identified an age-associated regulation of AGO2 mRNA stability. Aging Cell. 2018;17(3):e12753.

60. D'Aquila P, Bellizzi D, Passarino G. rRNA-gene methylation and biological aging. Aging (Albany NY). 2018;10(1):7-8.

61. Fang J, Hu M, Sun Y, et al. Expression Profile Analysis of m6A RNA Methylation Regulators Indicates They Are Immune Signature Associated and Can Predict Survival in Kidney Renal Cell Carcinoma. DNA Cell Biol. 2020.

62. Jasiulionis MG. Abnormal Epigenetic Regulation of Immune System during Aging. Front Immunol. 2018;9:197.

63. Bottazzi B, Riboli E, Mantovani A. Aging, inflammation and cancer. Semin Immunol. 2018;40:74-82.

64. Xu F, Zhang H, Chen J, et al. Immune signature of T follicular helper cells predicts clinical prognostic and therapeutic impact in lung squamous cell carcinoma. Int Immunopharmacol. 2020;81:105932.

65. Pan J, Huang Z, Xu Y. m5C RNA Methylation Regulators Predict Prognosis and Regulate the Immune Microenvironment in Lung Squamous Cell Carcinoma. Front Oncol. 2021;11:657466.

66. Wu Q, Xie X, Huang Y, et al. N6-methyladenosine RNA methylation regulators contribute to the progression of prostate cancer. J Cancer. 2021;12(3):682-92.

67. Zhang Y, Geng X, Li Q, et al. m6A modification in RNA: biogenesis, functions and roles in gliomas. J Exp Clin Cancer Res. 2020;39(1):192.

68. Li F, Zhang C, Zhang G. m6A RNA Methylation Controls Proliferation of Human Glioma Cells by Influencing Cell Apoptosis. Cytogenet Genome Res. 2019;159(3):119-25.

69. Fulop T, McElhaney J, Pawelec G, et al. Frailty, Inflammation and Immunosenescence. Interdiscip Top Gerontol Geriatr. 2015;41:26-40.

70. Coppe JP, Desprez PY, Krtolica A, et al. The senescence-associated secretory phenotype: the dark side of tumor suppression. Annu Rev Pathol. 2010;5:99-118.

71. Deng Y, Wang F, Hughes T, et al. FOXOs in cancer immunity: Knowns and unknowns. Semin Cancer Biol. 2018;50:53-64.

72. Vuong L, Kotecha RR, Voss MH, et al. Tumor Microenvironment Dynamics in Clear-Cell Renal Cell Carcinoma. Cancer Discov. 2019;9(10):1349-57.

73. Senbabaoglu Y, Gejman RS, Winer AG, et al. Tumor immune microenvironment characterization in clear cell renal cell carcinoma identifies prognostic and immunotherapeutically relevant messenger RNA signatures. Genome Biol. 2016;17(1):231.

74. Yuan L, Alexander PB, Wang XF. Cellular senescence: from anti-cancer weapon to anti-aging target. Sci China Life Sci. 2020;63(3):332-42.

**Figure Legends**

Figure 1. Differential expression and prognosis analysis of LUZP2.


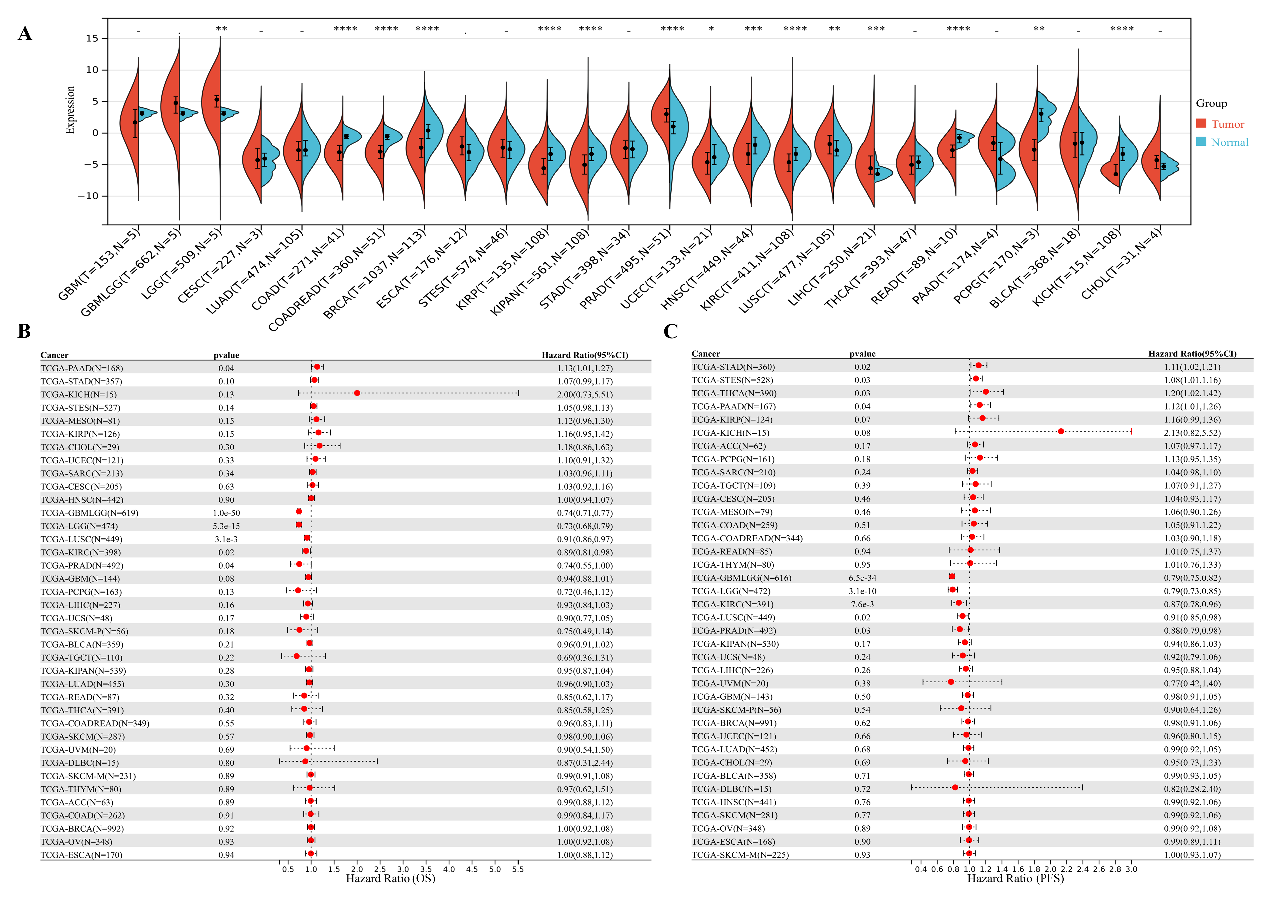


(A) pan-cancer analysis of LUZP2 for differential expression between tumor and normal tissues; (B) pan-cancer analysis of LUZP2 for OS; (C) pan-cancer analysis of LUZP2 for PFS. OS=overall survival; PFS=progression-free survival.

Figure 2. The pan-cancer Spearman analysis of tumor stemness and LUZP2 expression.


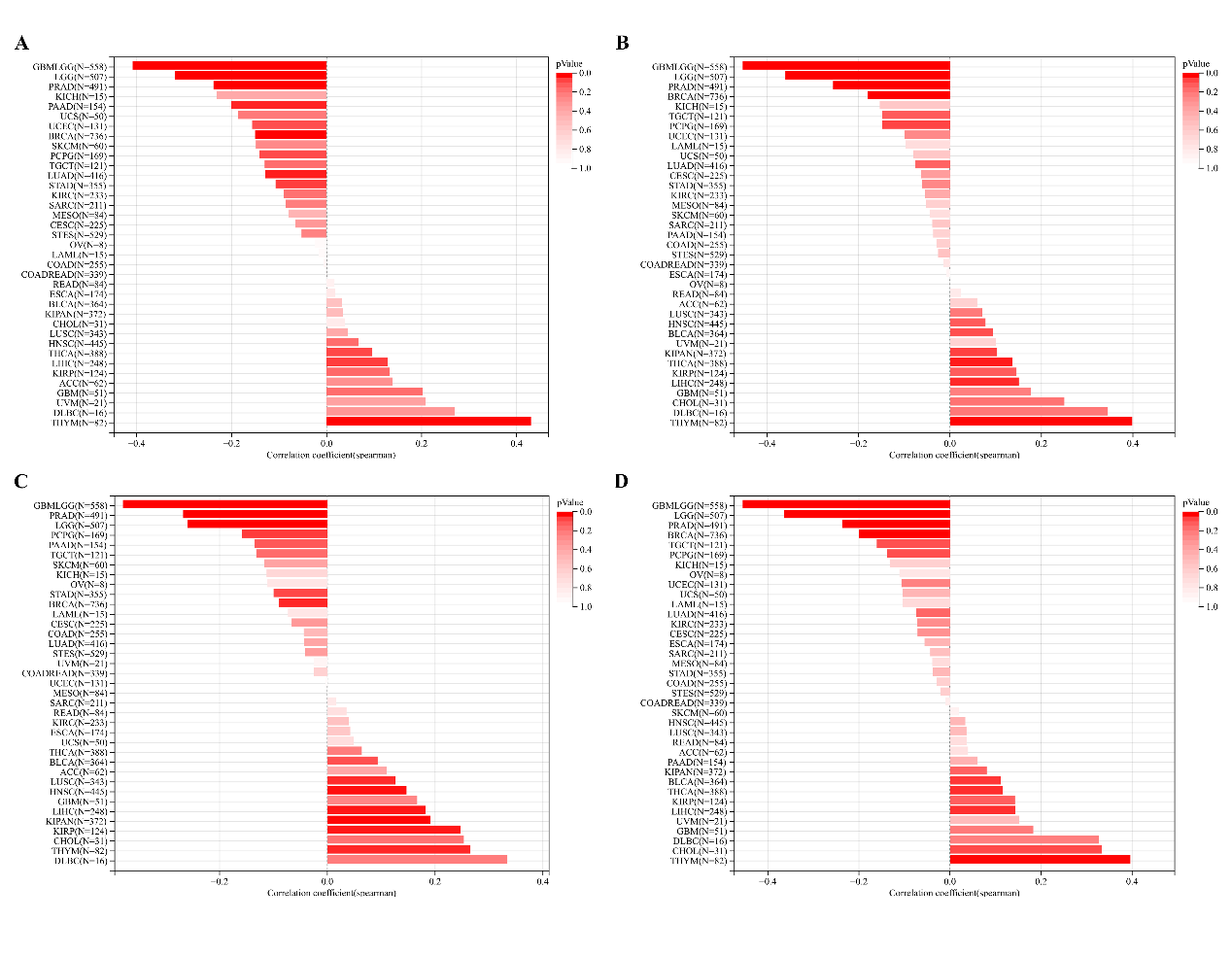


(A) the correlation between tumor stemness and LUZP2 level using DMPss; (B) the correlation between tumor stemness and LUZP2 level using DNAss; (C) the correlation between tumor stemness and LUZP2 level using ENHss; (D) the correlation between tumor stemness and LUZP2 level using EREG-METHss.

Figure 3. The pan-cancer Spearman analysis of tumor heterogeneity and LUZP2 expression.


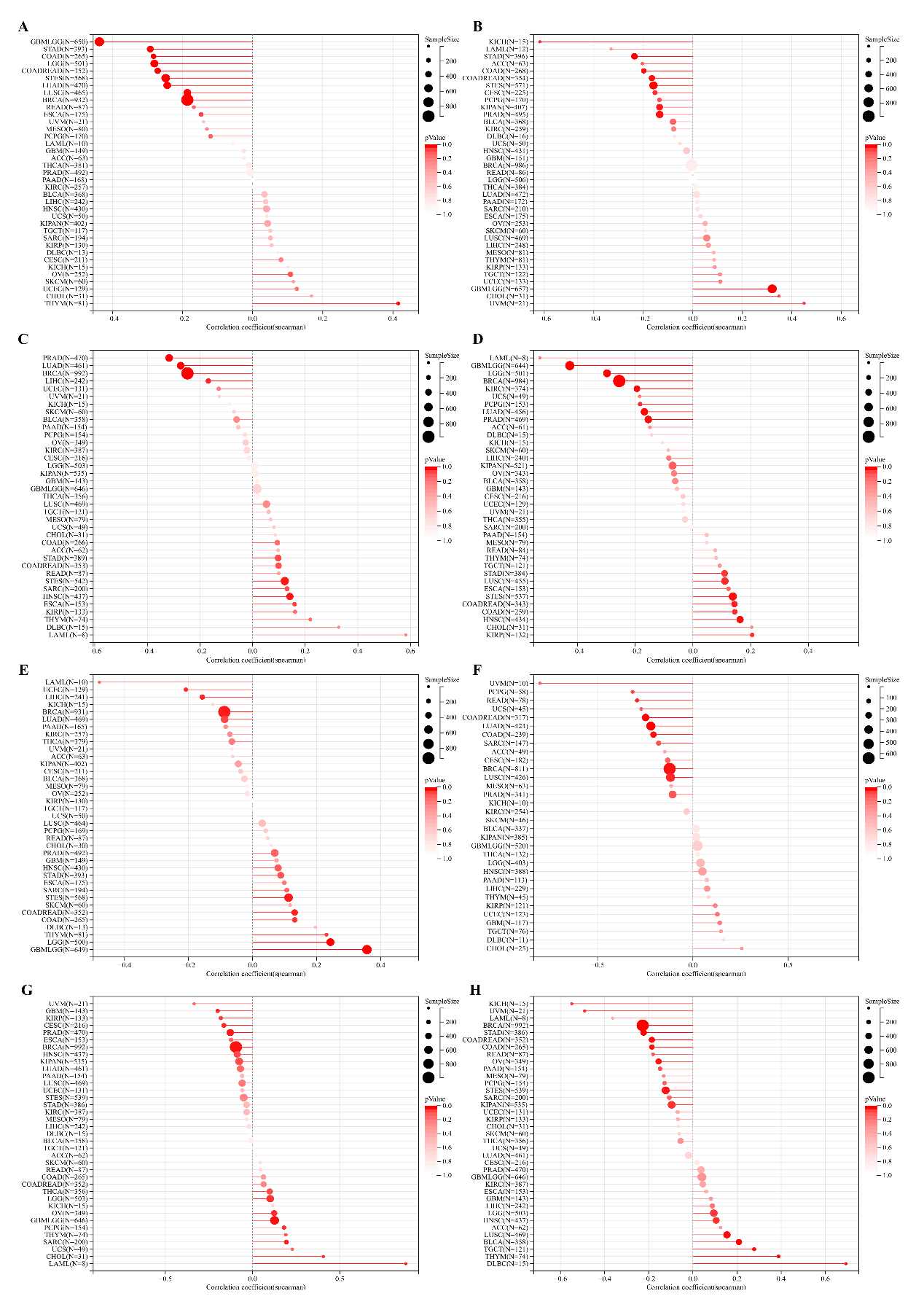


(A) the correlation between TMB and LUZP2 level; (B) the correlation between MSI and LUZP2 level; (C) the correlation between HRD and LUZP2 level; (D) the correlation between LOH and LUZP2 level; (E) the correlation between MATH and LUZP2 level; (F) the correlation between NEO and LUZP2 level; (G) the correlation between tumor ploidy and LUZP2 level; (H) the correlation between tumor purity and LUZP2 level. TMB=tumor mutation burden; MSI= microsatellite instability; HRD= homologous recombination deficiency; LOH= loss of heterozygosity; MATH= mutant-allele tumor heterogeneity; NEO= neoantigen.

Figure 4. Mutation landscapes analysis of LUZP2 and RNA modification.


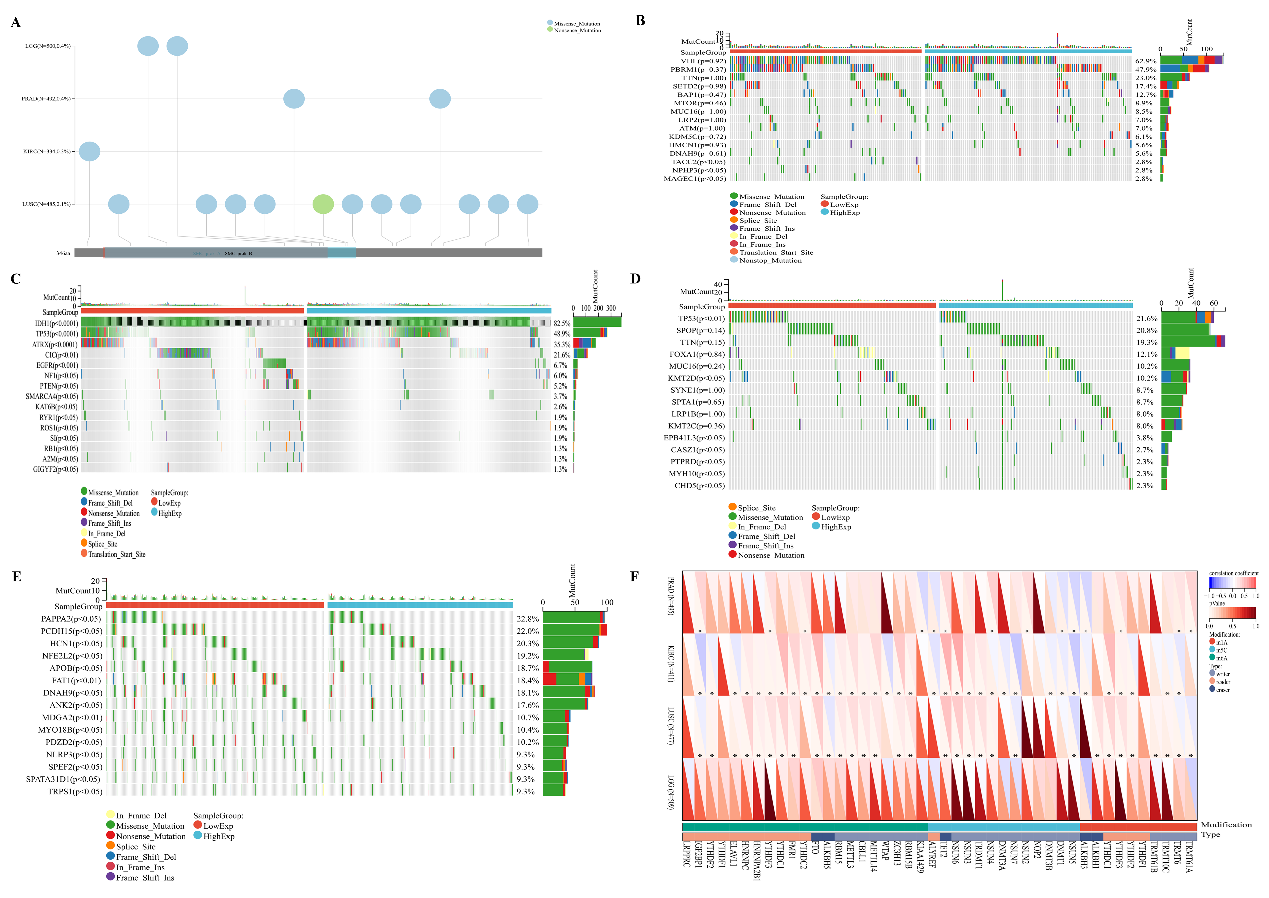


(A) mutation landscapes of LUZP2 for LGG, KIRC, LUSC, and PRAD; (B) the top 15 mutation genes between high and low-expression of LUZP2 in KIRC patients; (C) the top 15 mutation genes between high and low-expression of LUZP2 in LGG patients; (D) the top 15 mutation genes between high and low-expression of LUZP2 in PRAD patients; (E) the top 15 mutation genes between high and low-expression of LUZP2 in LUSC patients; (F) the correlation of LUZP2 expression and RNA modification genes.

Figure 5. Tumor immune environment and its correlation with LUZP2 methylation.


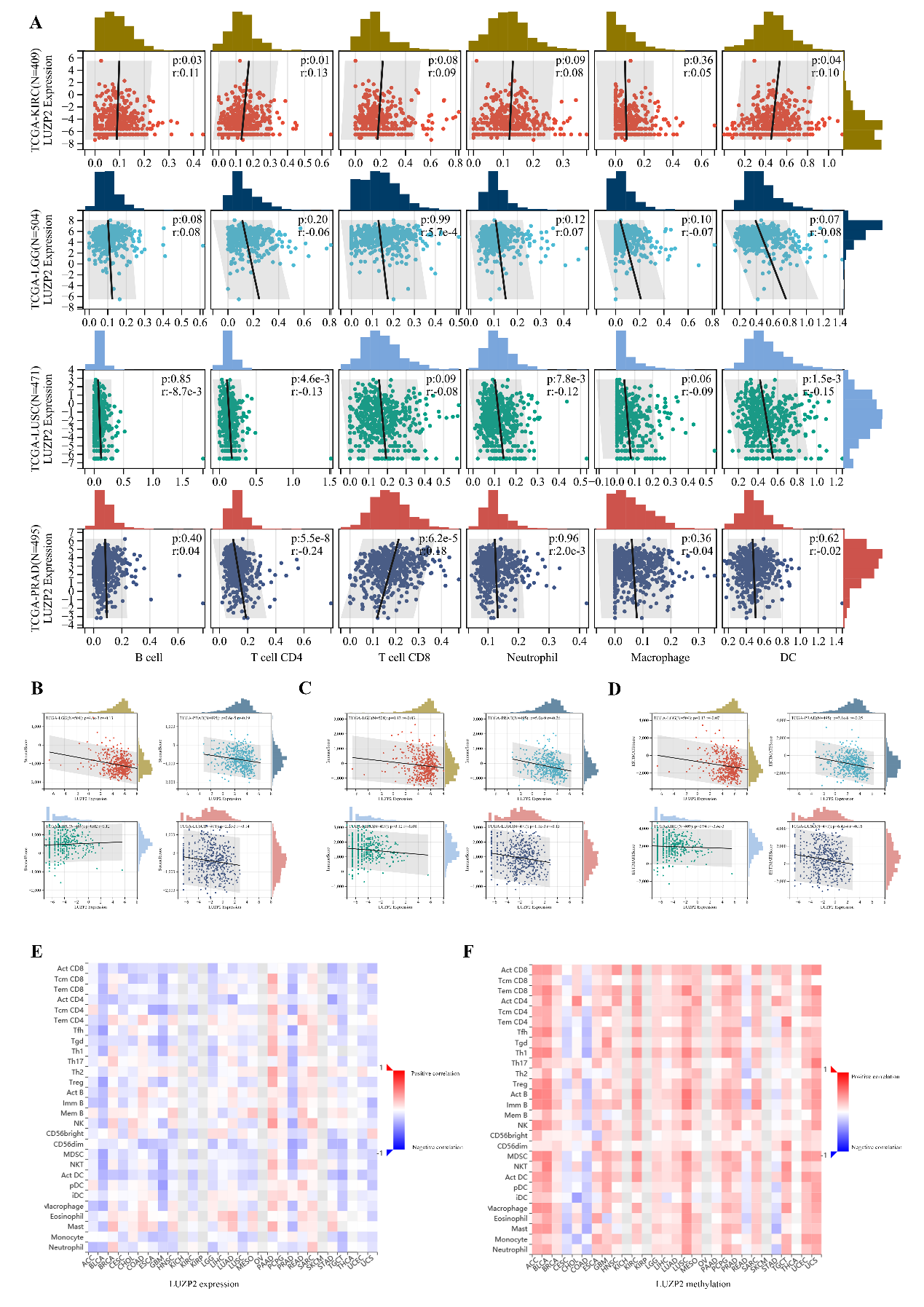


(A) the correlation of LUZP2 expression with immune cells; (B) the correlation of LUZP2 expression with stromal score; (C) the correlation of LUZP2 expression with immune score; (D) the correlation of LUZP2 expression with estimate score; (E) the correlation of LUZP2 expression with immune infiltrating cells in the TISIDB database [23]; (F) the correlation of LUZP2 methylation with immune infiltrating cells in the TISIDB database [23]. DC=dendritic cells.

Figure 6. The Spearman analysis of LUZP2 expression and immune checkpoints and regulatory genes.


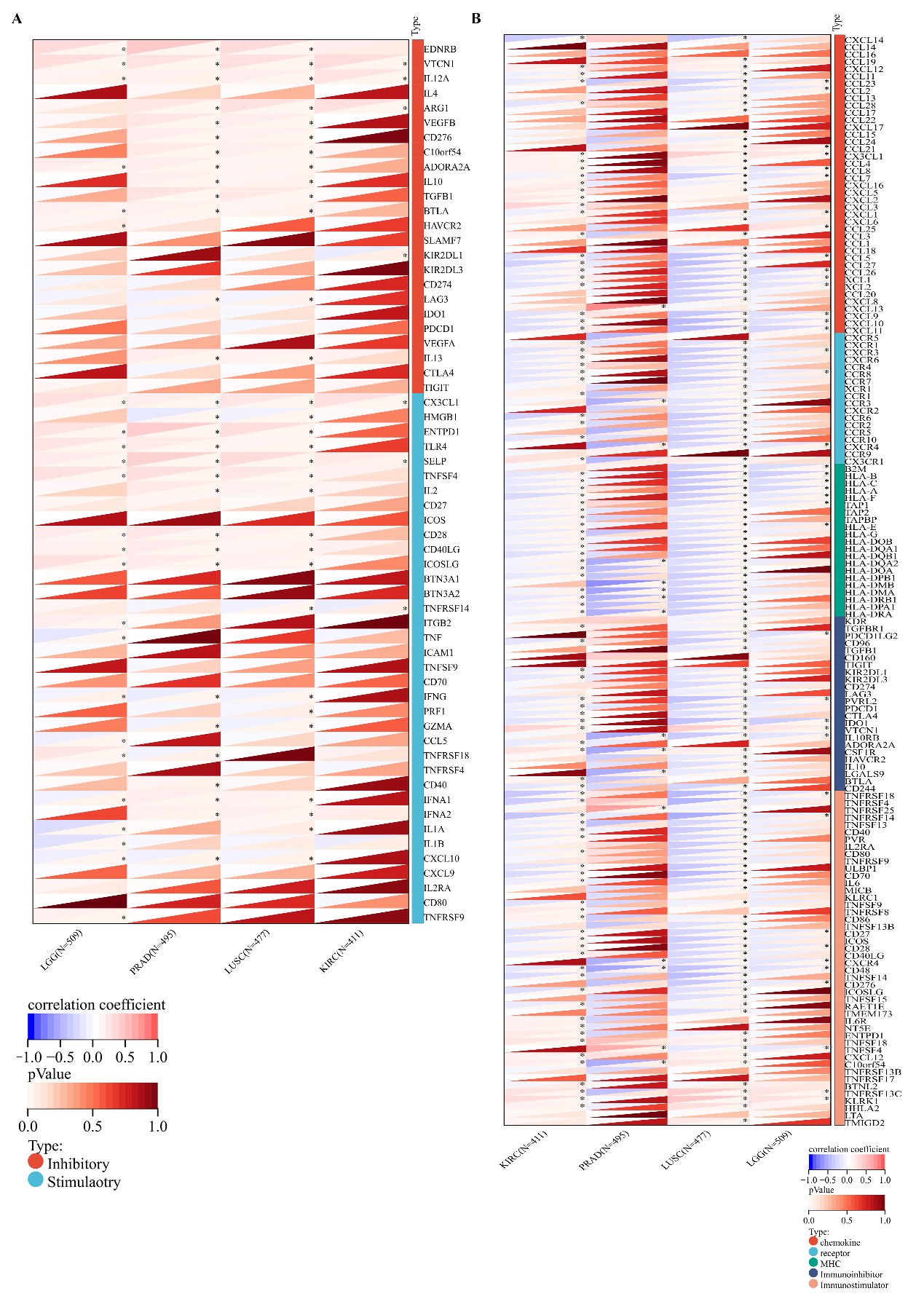


(A) the correlation of LUZP2 expression with immune checkpoints; (B) the correlation of LUZP2 expression with immune regulatory genes.

Supplementary figure 1. The pan-cancer analysis of clinical correlation with LUZP2 expression. (A) the correlation of LUZP2 expression with age; (B) the correlation of LUZP2 expression with gender; (C) the correlation of LUZP2 expression with grade; (D) the correlation of LUZP2 expression with T stages; (E) the correlation of LUZP2 expression with M stage; (F) the correlation of LUZP2 expression with N stages; (G) the correlation of LUZP2 expression with clinical stages.

Supplementary figure 2. The pan-cancer Spearman analysis of LUZP2 expression and gene mutations. (A) the correlation of LUZP2 expression with simple nucleotide variation; (B) the correlation of LUZP2 expression with copy number variation.
